# Not Opiates but Antiphlogistine

**Published:** 1903-06

**Authors:** 


### Not Opiates But Antiphlogistine.

Pain is the greatest instrument of torture with which the practitioner has to contend. It is the one symptom to which the laity attach the utmost importance. Absence of pain is to the patient always suggestive of improvement. Its presence, especially in uterine affections, causes apprehension of operation, and for relief of those cases who will not submit to operation and in inoperable conditions, Antiphlogistine strongly recommends itself, not only as a palliative measure but an excellent remedial agent. This fact has been successfully demonstrated by the gynecologist. Its value in acute and chronic conditions of the ovary and uterus is prompt, permanent and certain.

Two different methods of application are permissible, each exercising a distinct function in therapeutics.

During menstruation the introduction of any medicinal agent into the vagina is contra-indicated and at this period the pain of catamenial irregularities can best be controlled by applying Antiphlogistine over the abdomen warm and thick, and covering with cotton and a compress. This practice persisted in for several periods prevents headache, lumbar pain and other vicarious concomitant symptoms. Many women who have been physically incapacitated for a day or two each month have been permanently relieved by systematic use of Antiphlogistine at each menstrual illness. A potent influence is exerted over the sympathetic system which is so intimately associated with the physiological functions of the uterus that efferent stimulation neutralizes efferent irritation.

In the interval between menses, Antiphlogistine is successfully applied to the cervix of the uterus in the following manner: Make a small gauze sack and fill it with Antiphlogistine slightly larger in volume than the ordinary cotton tampon. Tie a string around the improvised sack and pass the Antiphlogistine tampon with dressing forceps through the vaginal speculum to the os of the uterus, molding around the cervix. Through the induction of osmosis and dialysis of inter-cellular fluid, intra-mural tension is quickly reduced, local analgesia and undisturbed cervical drainage follow. For relief of a patulous uterus, the indurated cervix of endometritis and all irregularities of menstruation, including amenorrhea and dysmenorrhea, this treatment is far superior to the ordinary glycerine tampon, rendering marvelous results to the clinician and patient.

